# Long noncoding RNA TANCR promotes γδ T cells activation by regulating TRAIL expression in *cis*

**DOI:** 10.1186/s13578-020-00383-6

**Published:** 2020-02-12

**Authors:** Chuan Yang, Ting Feng, Fang Lin, Tinxiang Gong, Shuo Yang, Yuhong Tao, Hong Li

**Affiliations:** 1 Key Laboratory of Birth Defects and Related Diseases of Women and Children of Ministry of Education, West China Second University Hospital, Sichuan University, No. 17, South Renmin Rd, Chengdu, 610000 China; 2 Chengdu Blood Center, No. 3, East Yvjie Rd, Chengdu, 61000 China; 3 Department of Pediatrics, West China Second University Hospital, Sichuan University, No. 20, South Renmin Rd, Chengdu, 610000 China

**Keywords:** LncRNA, γδ T cells, TRAIL, Immune cells, TANCR

## Abstract

**Background:**

γδ T cells are an important subset of T lymphocytes that play important roles in innate and adaptive immunity via the secretion of various cytokines. Previous studies have found that long noncoding RNAs (lncRNAs) are critical regulators that contribute to the development of immune cells. However, the functions of lncRNAs in the γδ T cells remains poorly studied.

**Results:**

Here, we identified the novel function of lncRNA NONHSAT196558.1 in isopentenyl pyrophosphate (IPP)-activated and -expanded γδ T cells using RNA-seq. As it functioned as an activating noncoding RNA of tumor necrosis factor related apoptosis-inducing ligand (TRAIL), an important cytotoxic cytokine that expressed by γδ T cells in responding to various infectious agents, we named this lncRNA as TANCR. Secondly, the expression of TANCR was found to be positively correlated with TRAIL expression in IPP activated γδ T cells. In addition, TANCR was confirmed to localized both in nucleus and cytoplasm. Finally, a loss-of-function was conducted by using siRNA/ASO or CRISPR/Cas9 system to knockdown or knockout TANCR, and confirmed that silencing of TANCR inhibits TRAIL expression in several kinds of cells, including HEK293T cells, Jurkat cells, and primary γδ T cells.

**Conclusion:**

These evidences demonstrate that TANCR play important roles in γδ T cell activation. Furthermore, TANCR may be involved in the cytotoxicity of γδ T cells. This study aims to further our understanding of the molecular mechanisms underlying lncRNA-mediated immune responses.

## Background

**Fig. 1:**
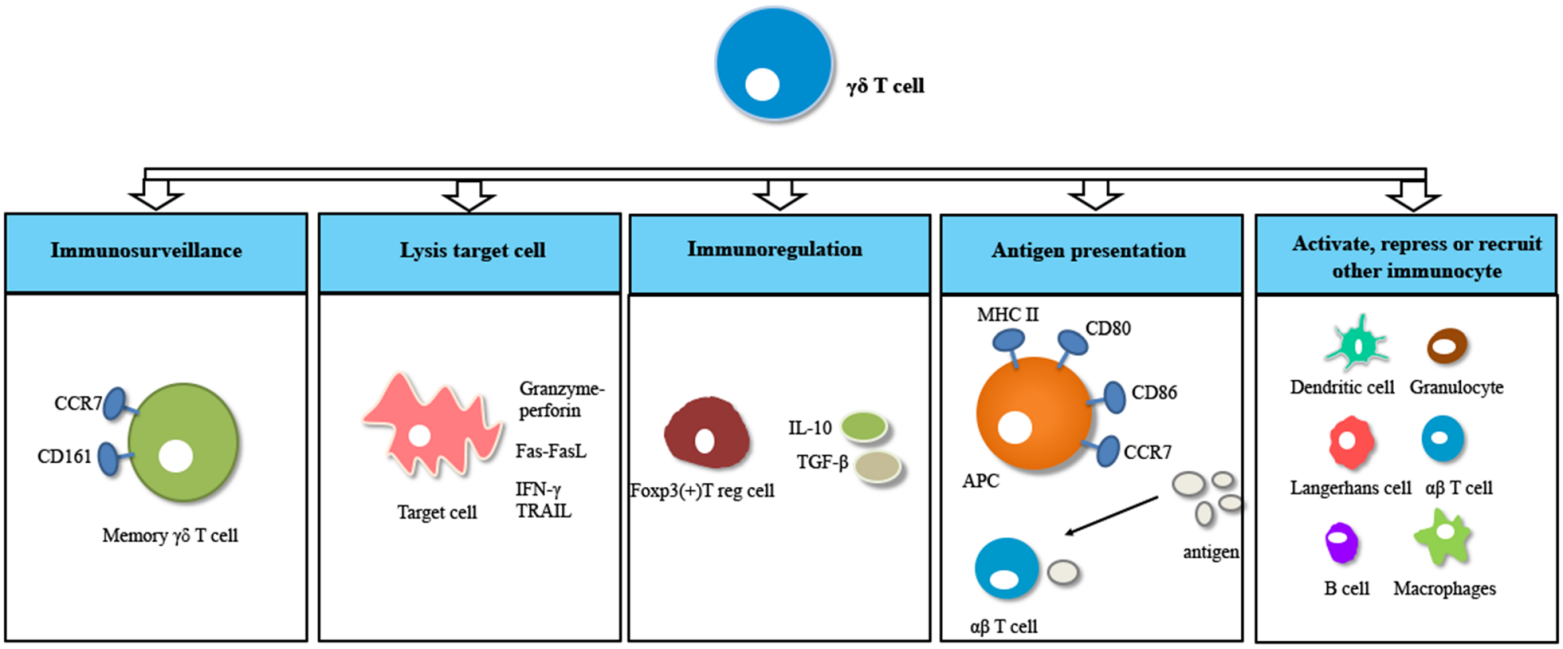
Functions of γδ T cells. γδ T cells differentiate into memory γδ T cells and function as immunosurveillance cells. Target cells were lysed by γδ T cell through granzyme-perforin, Fas–FasL, IFN-γ and TRAIL. γδ T cells can regulate Foxp3+ T-reg cell proliferation and secrete IL-10 and TGF-β during an immune response. γδ T cells can also differentiate into APCs and present antigens to αβ T cells. γδ T cells can activate, repress, or recruit other immunocytes during an immune response

γδ T cells are a subset of T lymphocytes that are classified as CD3 and T cell receptor (TCR) γδ double positive [1, 2]. γδ T cells occupy 5–10 percentage of peripheral T cells, while γδ T cells can be as high as 50% of total CD3^+^ T cells in some mucosal tissues [3, 4]. γδ T cells play critical roles in defending against various infections. On one hand, γδ T cells lyse target cells via granzyme-perforin secretion [5, 6]. On the other, they also trigger apoptosis of target cell through factor associated suicide (Fas)-Fas ligand (FasL), interferon-γ (IFN-γ) and TRAIL. Furthermore, γδ T cells can recruit other immune cells like dendritic cells, granulocytes, and langerhans cells to increase their anti-infection ability [7–9]. γδ T cells can differentiate into antigen presenting cells (APCs) and present antigens to αβ T cells [10]. In addition, γδ T cells modulate an immune response by regulating Foxp3( +) T reg cells proliferation and secreting interleukin 10 (IL-10) and transforming growth factor β (TGF-β) [11]. The functions of γδ T cells are showed in Fig. 1 (edited according to reference [12]).

It is well known that the majority of the genome transcribes noncoding genes and the functions of various noncoding genes are unknown [13]. Micro-RNAs are a well-known noncoding RNA type that regulates gene expression by binding the 3′ untranslated region of a target gene [14]. LncRNA is transcript that is longer than 200 nucleotides [13]. LncRNAs modulate gene expression via binding to proteins or interacting with DNA/RNA directly [15–17]. LncRNA mediation of cis regulation involves neighboring genes that transcribed from the same allele. While when lncRNA exerts its influence on a distantly located genes, lncRNA act in trans [18]. Previous studies have showed the evidence that lncRNA regulate various genes in the immune system. For example, the lncRNA NeST controls the expression of the interferon-γ locus in response to bacterial infection [19]. LncRNA lnc-DC regulates the differentiation of dendritic cell via binding to signal transducer and activator of transcription 3 [20]. Long intergenic ncRNA EPS acts as an inhibitor in the inflammatory response in macrophages [21]. Taken together, these evidences strongly indicate that lncRNAs act as critical regulators in the immune system. However, there are few studies about lncRNA regulation in γδ T cells. Hence, exploring the lncRNA regulation network in γδ T cells may help us better understand the function of lncRNA in γδ T cell biology.

IPP is a mevalonate pathway product that can selectively activate and expand human γδ T cells [22–24]. In this study, γδ T cells were activated by IPP, and then we investigated the differentially expressed genes including lncRNAs and mRNAs in the activated of γδ T cells compared with fresh γδ T cells using RNA-seq. Bioinformatic analysis was performed to predict lncRNA–mRNA pairs that regulated in cis, we found that the lncRNA NONHSAT196558.1 (named as TANCR [25]) expressed much higher in IPP-activated γδ T cells, and positively correlated with tumor necrosis factor related apoptosis-inducing ligand (TRAIL, also known as TNFSF10) expression. Using siRNA/ASO or CRISPR/Cas9 system to knockdown or knockout TANCR confirmed that silencing of TANCR inhibits TRAIL expression. The current results demonstrate that TANCR positively regulates TRAIL expression in the activation of γδ T cells, which provide epigenetic mechanisms in understanding lncRNAs in γδ T cells.

## Results

### Expansion of γδ T cells

**Fig. 2:**
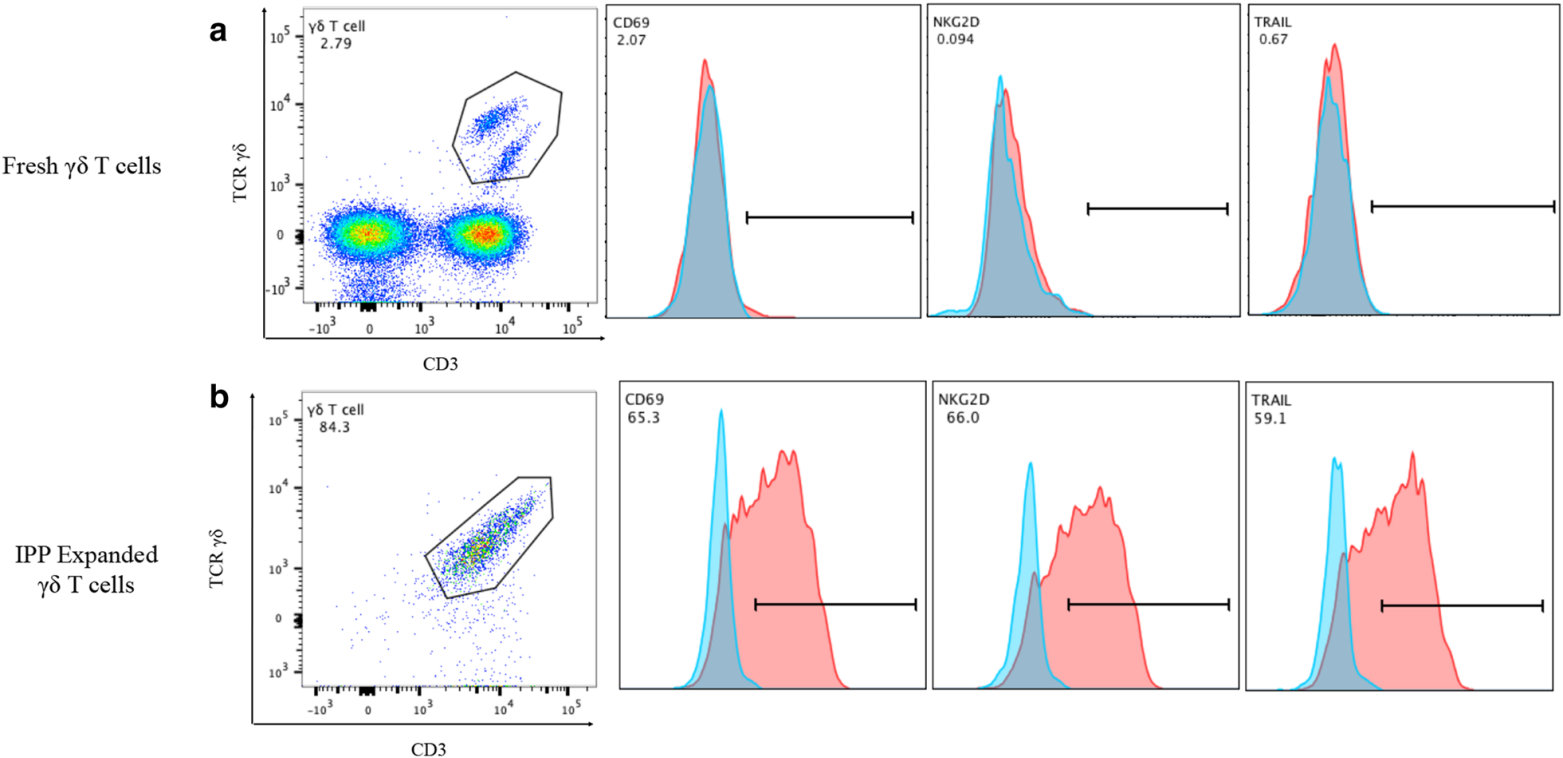
Phenotypes of fresh and IPP-expanded γδ T cells. The percentage of γδ T cells and expression of CD69, NKG2D, and TRAIL in fresh γδ T cells (**a**) and IPP-expanded γδ T cell (**b**)

To obtain activated γδ T cells, IPP was used to activate γδ T cell. Freshly isolated (peripheral blood mononuclear cells) PBMCs were obtained from healthy adult donors. After three weeks of culture in the presence of IL-2 and IPP, the percentage of γδ T cells within CD3 cells increased to 84.3% (Fig. 2b). To determine whether γδ T cells were activated by IPP, we examined cell surface markers (CD69, NKG2D, and TRAIL) in fresh and IPP expanded γδ T cells. As shown in Fig. 2, fresh γδ T cells expressed low levels of CD69, NKG2D, and TRAIL. In contrast, IPP expanded γδ T cells expressed much higher levels of CD69, NKG2D, and TRAIL compared with fresh γδ T cells (Fig. 2a, b). Then fresh and IPP expanded γδ T cells were purified with MACS, respectively. RNA was extracted and sent for RNA-seq.

### Characteristics of differentially expressed lncRNAs and mRNAs in IPP-expanded γδ T cells

**Fig. 3:**
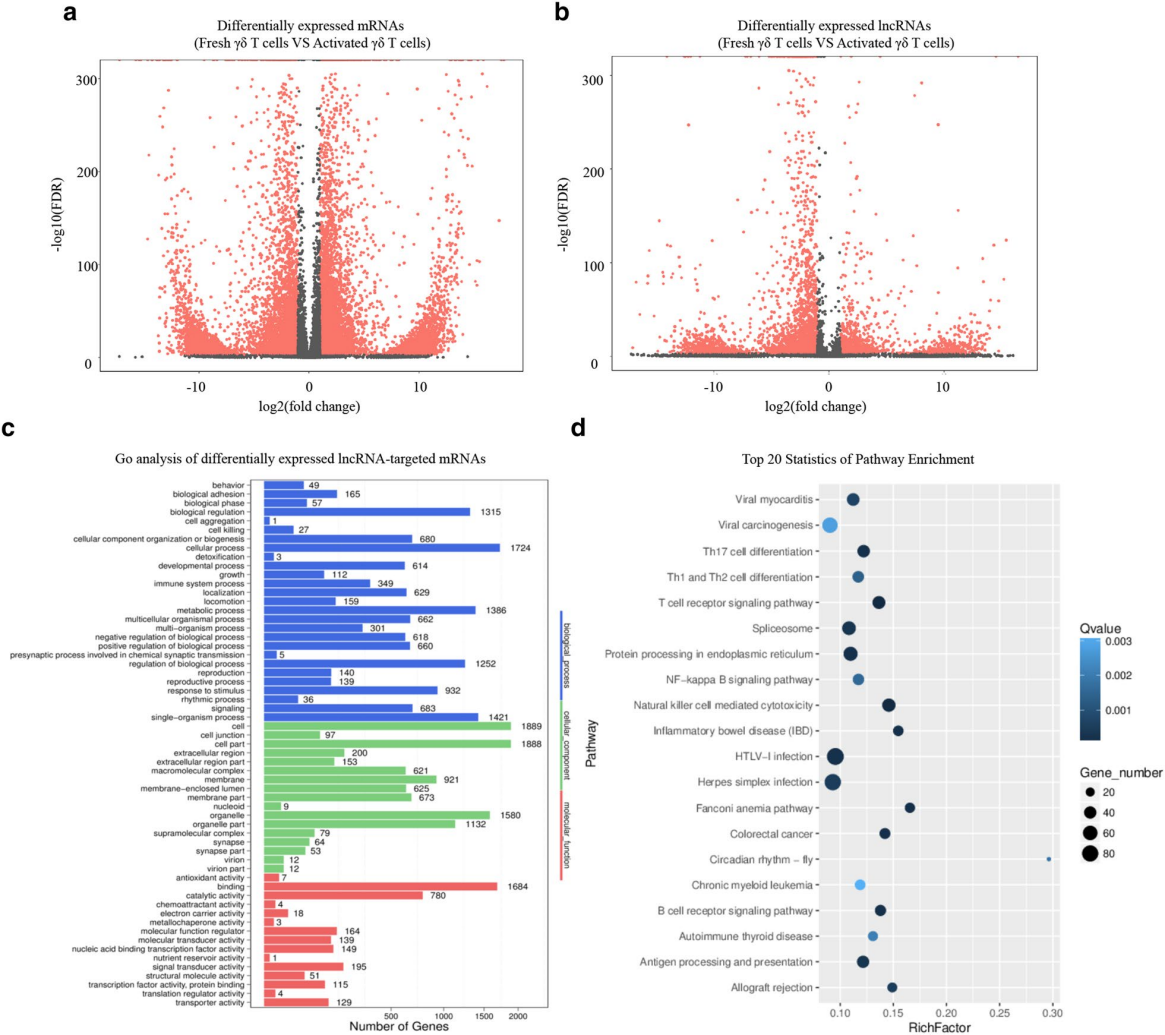
Gene expression profiles in activated and fresh γδ T cells. **a** Volcano plots of differentially expressed mRNAs. **b** Volcano plots of differentially expressed lncRNAs. The red symbols in volcano plots represent significantly upregulated or downregulated genes (fold change ≥ 2, p < 0.05). **c** Go analysis of differentially expressed lncRNA-targeted mRNAs. Biological processes, cellular components, and molecular functions are shown. **d** KEGG pathway analysis of differentially expressed lncRNA-targeted mRNAs. The top 20 enriched pathways are shown

In order to explore the lncRNA–mRNA pairs that regulated in cis in γδ T cell activation, a PossionDis assay was used to analyze the differentially expressed mRNA and lncRNA. In total, 18,183 mRNAs and 5378 lncRNAs were found to be differentially expressed (fold change > 2, p-value < 0.05). A volcano plot was used to display the differentially expressed mRNAs and lncRNAs (Fig. 3a, b). The results showed that the expression of mRNA and lncRNA in IPP-expanded γδ T cells was significantly different from the fresh γδ T cells, which indicates that there might be lncRNA-mRNA interactions in the differentially expressed genes. LncRNA-mRNA interactions are an important regulatory mechanism of lncRNAs. We then analyzed the *cis*-regulation of differentially expressed genes based on the co-localization and co-expression of the lncRNAs and mRNAs. The top 15 upregulated and downregulated lncRNAs and target mRNAs are shown in Table 1.

**Table 1:** Top 15 up and down regulated lncRNA and target mRNA

LncRNA ID	LncRNA location	Target mRNA	Control FPKM	Exp FPKM	FC	Up/down	p value
NONHSAT121988.2	cis_mRNA_overlap	NM_001145306	0.01	7.28	728	Up	3.77E‒70
NONHSAT137540.2	cis_mRNA_overlap	NR_003255	0.02	5.32	266	Up	1.23E‒294
NONHSAT152541.1	cis_mRNA_overlap	NM_003762	0.01	2.06	206	Up	6.51E‒18
NONHSAT137555.2	cis_mRNA_overlap	NR_003255	0.02	3.96	198	Up	4.72E‒99
NONHSAT182712.1	cis_mRNA_overlap	NM_014607	0.02	3.52	176	Up	9.26E‒26
NONHSAT114689.2	cis_mRNA_overlap	NM_001029858	0.86	137.81	160	Up	3.85E‒131
NONHSAT056185.2	cis_mRNA_dw20k	NM_001127198	0.04	5.72	143	Up	2.16E‒71
NONHSAT207077.1	cis_mRNA_overlap	NM_014046	0.12	15.01	125	Up	8.60E‒17
NONHSAT071221.2	cis_mRNA_overlap	NR_130931	0.14	12.9	92	Up	1.93E‒76
NONHSAT093397.2	cis_mRNA_dw20k	NM_033540	0.01	0.88	88	Up	0.0321706
NONHSAT172882.1	cis_mRNA_dw20k	NM_013258	0.33	28.68	87	Up	4.75E‒105
NONHSAT148955.1	cis_mRNA_overlap	NM_024646	0.01	0.83	83	Up	0.00683706
NONHSAT119974.2	cis_mRNA_overlap	NR_036501	0.01	0.74	74	Up	2.34E‒05
NONHSAT138724.2	cis_mRNA_overlap	NR_038461	0.09	6.45	72	Up	5.57E‒13
NONHSAT026084.2	cis_mRNA_dw20k	NM_001297438	0.02	1.37	68.5	Up	0.0001099758
NONHSAT103660.2	cis_mRNA_overlap	NM_001046	13.12	0.05	262.4	Down	0.001397956
NONHSAT176182.1	cis_mRNA_overlap	NM_000964	2.55	0.01	255	Down	2.58E‒08
NONHSAT179933.1	cis_mRNA_overlap	NM_001317113	31.62	0.13	243	Down	1.94E‒169
NONHSAT151799.1	cis_mRNA_overlap	NM_201624	4.11	0.02	205.5	Down	8.82E‒18
NONHSAT069808.2	cis_mRNA_overlap	NM_001329113	4.09	0.17	204.5	Down	2.11E‒30
NONHSAT041504.2	cis_mRNA_up10k	NR_036650	6.44	0.04	161	Down	0.000227306
NONHSAT096695.2	cis_mRNA_overlap	NR_015439	1.6	0.01	160	Down	5.78E‒19
NONHSAT176679.1	cis_mRNA_dw20k	NM_001316321	2.82	0.02	141	Down	1.04E‒08
NONHSAT119667.2	cis_mRNA_overlap	NR_038367	1.3	0.01	130	Down	9.17E‒05
NONHSAT097487.2	cis_mRNA_overlap	NM_001100426	1.29	0.01	129	Down	9.17E‒05
NONHSAT069824.2	cis_mRNA_overlap	NM_005253	236.63	1.96	121	Down	3.83E‒57
NONHSAT000029.2	cis_mRNA_overlap	NR_039983	2.34	0.02	117	Down	6.46E‒37
NONHSAT082319.2	cis_mRNA_dw20k	NM_004915	3.43	0.03	114	Down	8.57E‒27
NONHSAT141480.2	cis_mRNA_overlap	NM_001024401	4.46	0.04	111.5	Down	5.45E‒42
NONHSAT185717.1	cis_mRNA_dw20k	NR_147695	88	0.83	106	Down	4.41E‒156

**Fig. 4:**
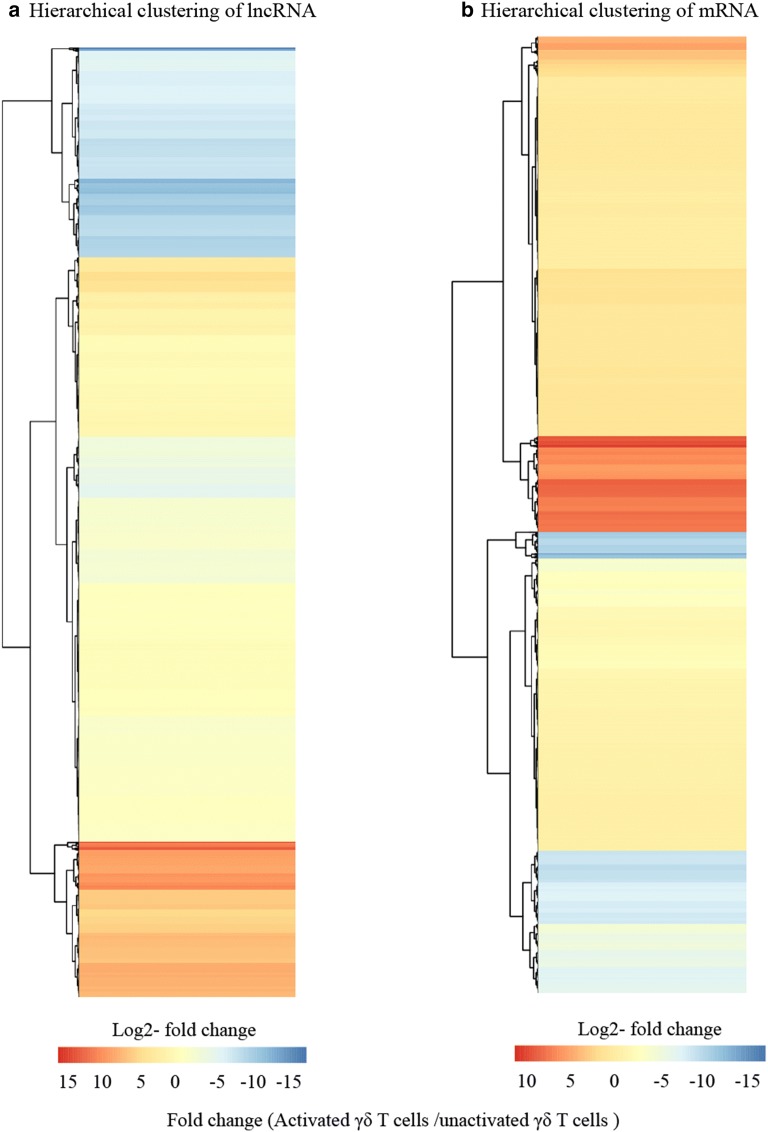
Hierarchical clustering of differentially expressed genes. **a** Hierarchical clustering of differentially expressed lncRNAs. **b** Hierarchical clustering of differentially expressed mRNAs. The color scale of the strips runs from red to blue, which means the gene was up-regulated or down-regulated. log2-fold change values were calculated from RNA-seq

To further study the function of differentially expressed lncRNA-targeted mRNAs in the activation of γδ T cells, Go and KEGG analysis were performed. Go analysis revealed that the most frequent terms were cellular process in biological process, cell in cellular component, and binding in molecular function (Fig. 3c). The KEGG results showed the 20 top enriched pathways (Fig. 3d). T cell receptor signaling pathway, Th17 cell differentiation, and Th1 and Th2 cell differentiation are included in the 20 top enriched pathways, which indicates that the differentially expressed lncRNA-targeted mRNAs may be involved in T cell activation. Genes including mRNA and lncRNA that were differentially expressed between IPP-expanded and fresh γδ T cells were then analyzed by a hierarchical cluster (fold change), which demonstrates that the genes were distinguishable between the two groups (Fig. 4a, b).

### TANCR is expressed highly in the nucleus and is positively correlated with TRAIL expression

**Fig. 5:**
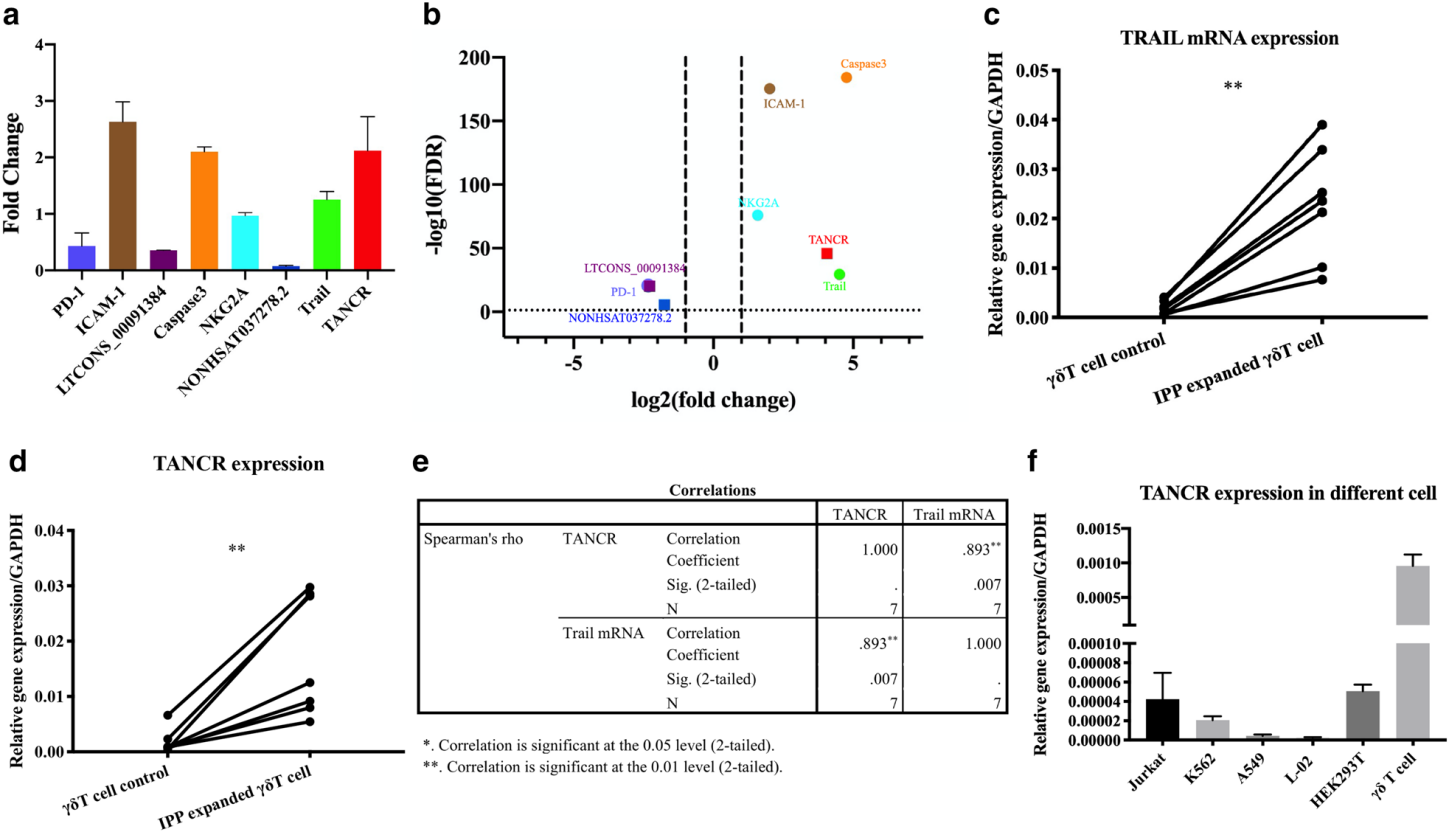
Expression of TANCR. **a** Validation of RNA-seq, qRT-PCR results of eight selected genes. **b** Volcano plots of eight selected genes in RNA sequencing results, thick vertical lines represent fold changes of ‒ 2 and + 2, while thick horizontal lines stand for negative log10 of FDR. TRAIL (**c**) and TANCR (**d**) expression in IPP-expanded γδ T cells, γδ T cell control represents γδ T cells without IPP treatment, qRT-PCR was normalized by GAPDH and p value was calculated by paired Student’s t-test, n = 7. **e** Spearman’s correlation analysis of TANCR and TRAIL mRNA expression. **f** TANCR expression in different cells. Data are represented as means ± SEM from three individual experiments, qRT-PCR was normalized by GAPDH

To validate the sequencing results, eight genes were selected to validate using reverse transcription polymerase chain reaction (RT-PCR), including mRNA and lncRNA (caspase3, intercellular cell adhesion molecule-1 (ICAM-1), programed death-1 (PD-1), natural killer cell receptor G2A (NKG2A), TRAIL, TANCR, NONHSAT037278.2, and LTCONS_00091384). The RT-PCR results were similar to those observed in sequencing results (Fig. 5a, b). TRAIL is considered as important cytokine that mediates γδ T cell cytotoxicity. In addition, bioinformatic analysis also showed that lncRNA TANCR may regulate TRAIL expression in cis. In order to test whether TANCR expression correlate with TRAIL expression, we treated PBMCs isolated from seven donors with IPP and detected the expression of TRAIL and TANCR in purified γδ T cells. qRT-PCR results showed that both TRAIL mRNA and TANCR were increased in IPP-expanded γδ T cells (Fig. 5c, d). The expression of TRAIL also correlated with TANCR expression (Fig. 5e). These results indicated that TANCR might regulate TRAIL mRNA expression in cis. In order to know whether TANCR specifically expressed in γδ T cells, we then performed qRT-PCR in six different cell types including Jurkat cell line, K562 cell line, A549 cell line, L-02 cell line, HEK293T cell line, and γδ T cells. Our results showed that TANCR is more highly expressed in γδ T cells than the other five cell types (Fig. 5f), suggesting that TANCR might be specifically expressed in γδ T cells and regulate TRAIL expression.

**Fig. 6:**
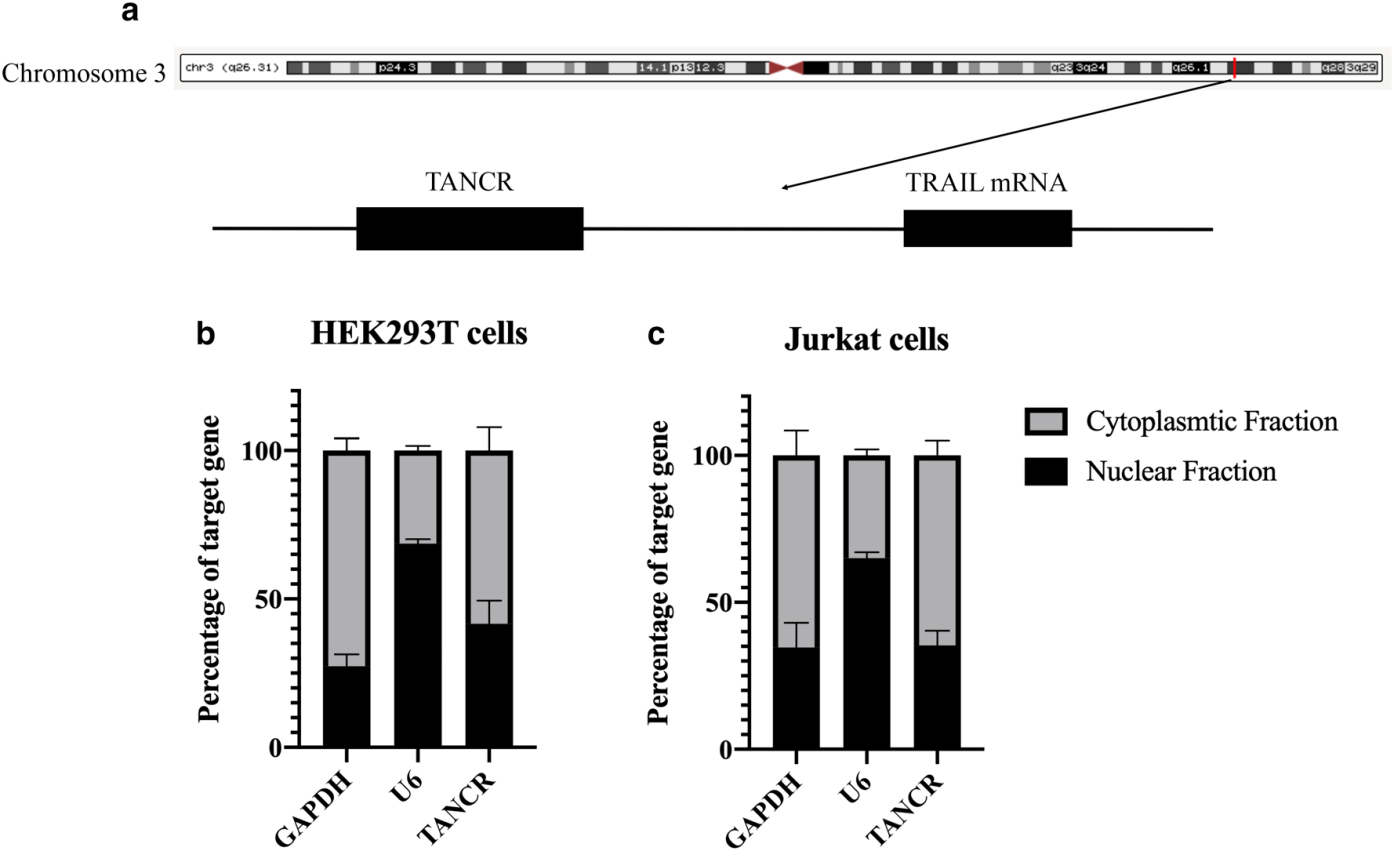
Relative location of TANCR and TRAIL in Chromosome 3 and cellular localization of TANCR. **a** Relative location of TANCR and TRAIL in Chromosome 3. **b** Cellular localization of TANCR in HEK293T cells. **c** Cellular localization of TANCR in Jurkat cells. GAPDH was used as cytoplasmic control and U6 was used as nuclear control. Graphs show the mean values ± SEM and data obtained from three independent experiments

TANCR is a transcript that located in the downstream of TRAIL mRNA in Chromosome 3 (Fig. 6a). To clarify the localization of TANCR, nuclear RNA and cytoplasmic RNA was extracted from HEK293T cell line and Jurkat cell line. GAPDH and U6 were used as cytoplasmic and nuclear control, respectively. qRT-PCR results showed that TANCR expressed both in the cytoplasm and nucleus, while TANCR is predominantly expressed in the cytoplasm and partially expressed in the nucleus (Fig. 6b, c).

### Overexpression/knockdown of TANCR in HEK293T cells affects TRAIL expression

**Fig. 7:**
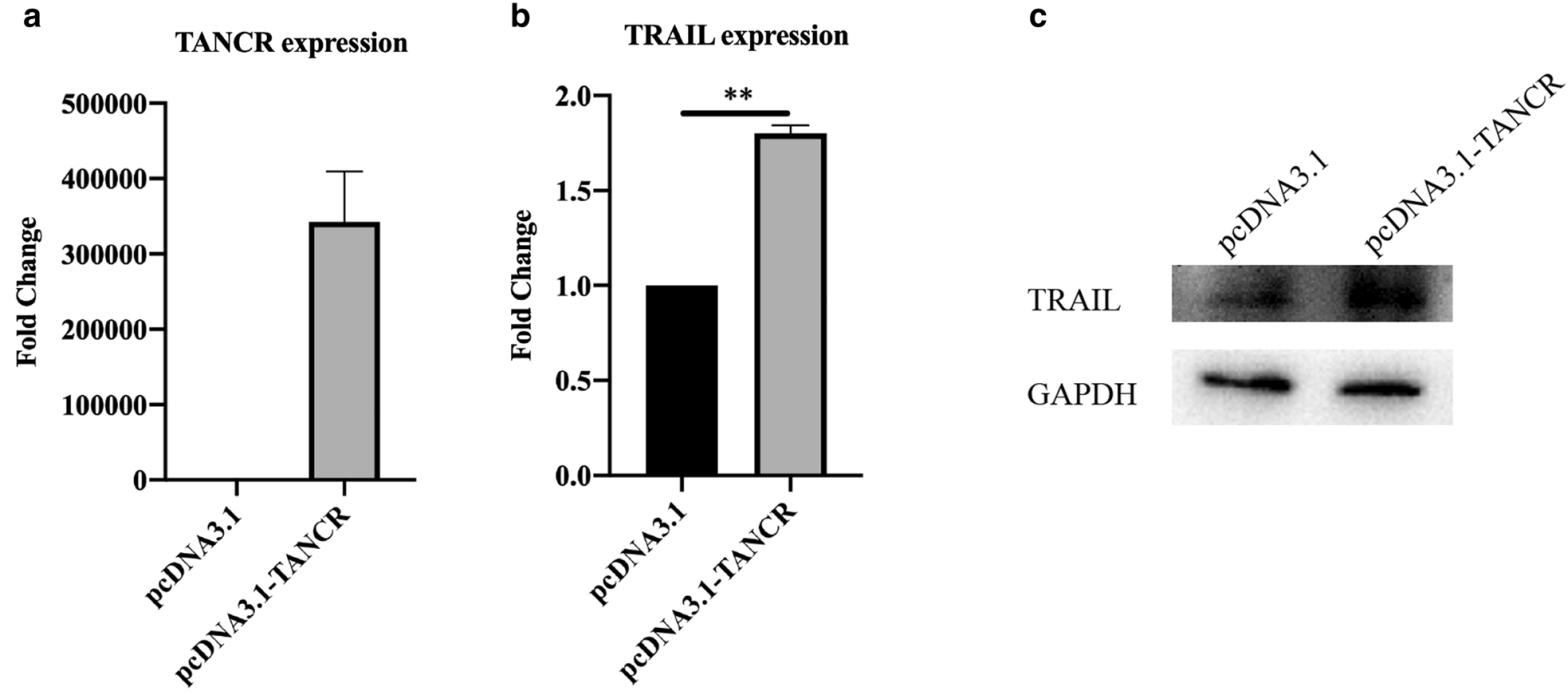
Overexpression of TANCR in HEK293T cells. TANCR (**a**) and TRAIL (**b**) mRNA expression in TANCR overexpressed cells. **c** Western blot of TRAIL in TANCR overexpressed HEK293T cells. qRT-PCR and western blot were normalized by GAPDH. Graphs show the mean values ± SEM and data obtained from three independent experiments. p value is calculated by Student’s t-test

**Fig. 8:**
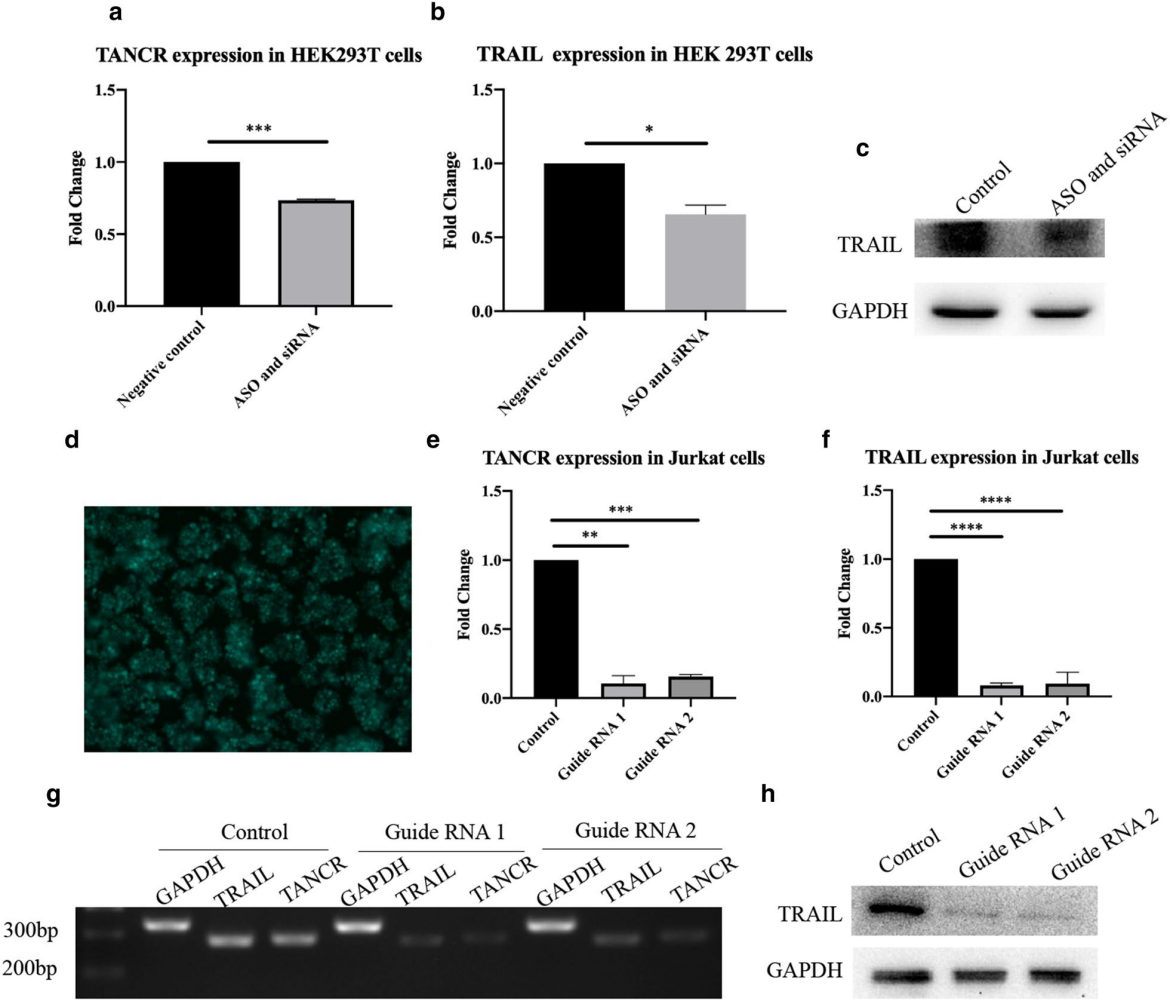
Silencing of TANCR in HEK293T cells and Jurkat cells. **a** qRT-PCR results of TANCR expression in TANCR knockdown HEK293T cells. **b** qRT-PCR results of TRAIL mRNA expression in TANCR knockdown HEK293T cells. **c** Western blot of TRAIL protein in TANCR knockdown HEK293T cells. **d** Expression of Cas9 in Jurkat cells. Two vector CRISPR/Cas 9 system was used in this study, the first vector contains cas9 and GFP and the second vector contains gRNA. **e** RT-PCR results of TANCR expression in TANCR knock out Jurkat cells. **f** qRT-PCR results of TRAIL mRNA expression in TANCR knock out Jurkat cells. **g** Agarose gel electrophoresis of qRT-PCT results. **h** Western blot of TRAIL protein in TANCR knock out Jurkat cells. qRT-PCR and western blot were normalized by GAPDH. Graphs show the mean values ± SEM and data obtained from three independent experiments. p value is calculated by Student’s t-test

To confirm whether TANCR affects TRAIL expression, we constructed pcDNA3.1-TANCR vector and transfected it into HEK293T cells. The RT-PCR and Western blot results showed that TRAIL expression was increased both in mRNA and protein level in TANCR overexpressed HEK293T cell (Fig. 7a, b). Small interfering RNAs (siRNA) and antisense oligonucleotides (ASO) were then transfected into HEK293T cells to knockdown TANCR expression in both cytoplasm and nucleus based on the localization of TANCR explored above. The RT-PCR results showed that siRNA and ASO can reduce the expression of TANCR (Fig. 8a). With the downregulation of TANCR, both mRNA and protein expression of TRAIL was decreased (Fig. 8b, c).

### Loss of TANCR abolishes TRAIL expression in Jurkat cells and primary γδ T cell

**Fig. 9:**
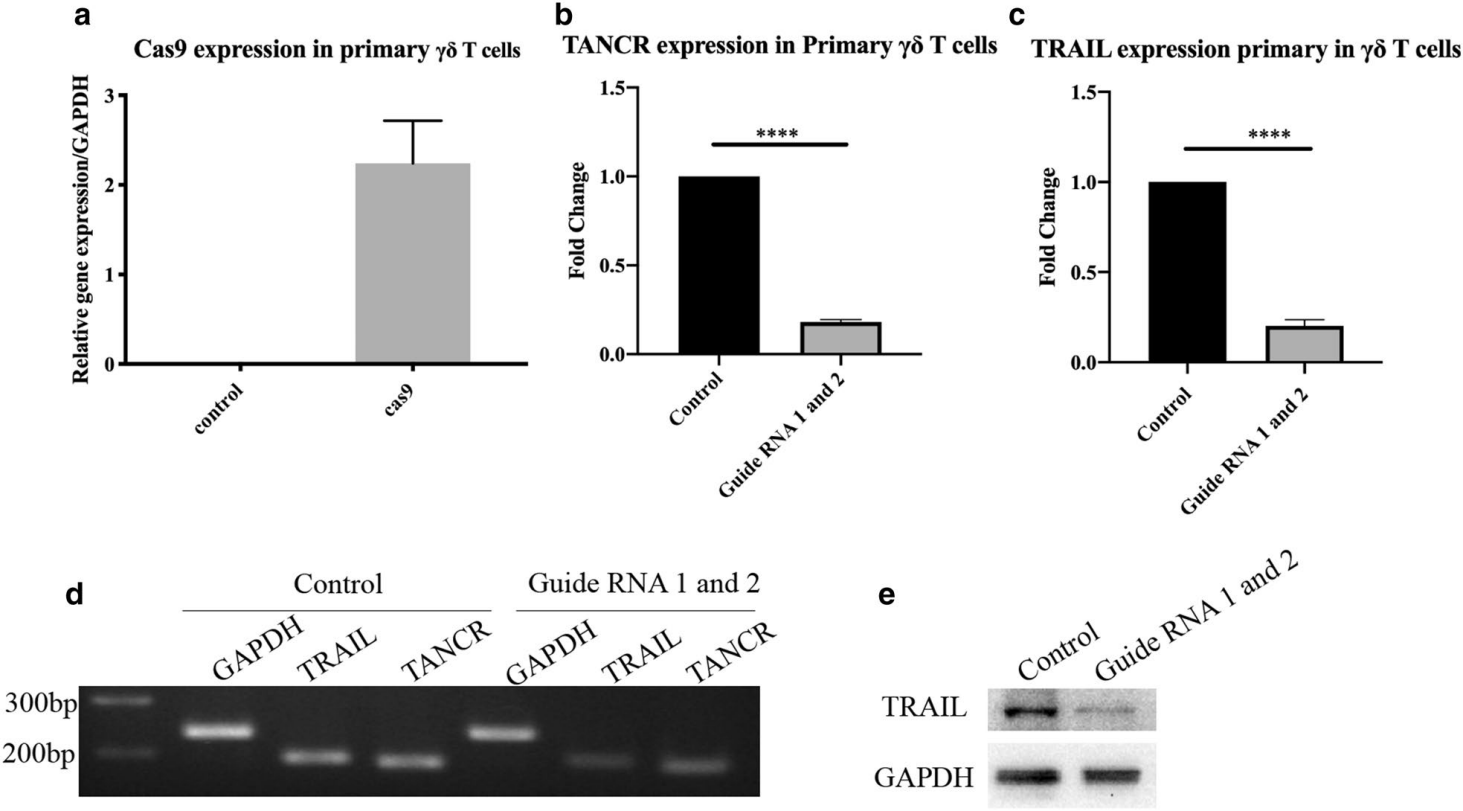
Silencing of TANCR in primary γδ T cells. **a** Cas9 expression in primary γδ T cells. **b** Expression of TANCR in TANCR KO primary γδ T cells, a guide RNA mixture (containing guide RNA 1 and guide RNA 2 used in Jurkat cells) was transduced into primary γδ T cells. **c** Expression of TRAIL mRNA in TANCR KO primary γδ T cells. **d** Agarose gel electrophoresis of qRT-PCR results. **e** Western blot of TRAIL in TANCR KO primary γδ T cells. qRT-PCR and western blot were normalized by GAPDH. Data are represented as means ± SEM from three individual experiments. p value is calculated by Student’s t-test

To further confirm the regulation between and TANCR and TRAIL, CRISPR/Cas 9 system was used to knock out TANCR expression in Jurkat cells and primary γδ T cells. CRISPR/Cas 9 vector with GFP was first transduced into Jurkat cells, and the cells expressed high level of Cas 9 (Fig. 8d). Two guide RNAs were then transduced into Jurkat cells with lentivirus system. The results showed that both two guide RNAs could effectively reduce the expression of TANCR in Jurkat cells (Fig. 8e). And a correlation of a reduced expression of TRAIL mRNA and protein was observed with a loss of TANCR expression in Jurkat cells (Fig. 8f, h). Electrophoresis of RT-PCR results also confirmed the downregulation of TRAIL mRNA in the absence of TANCR (Fig. 8g). A vector with Cas9 was first transduced into primary γδ T cells (Fig. 9a). Then a guide RNA mixture (containing guide RNA 1 and guide RNA 2 used in Jurkat cells) with lentivirus was transduced into primary γδ T cells obtained from PBMCs (Fig. 9b). Our result showed that the expression of TANCR was decreased, resulting in a reduction in the expression of TRAIL mRNA and protein (Fig. 9c–e), which consistent with the results in HEK293T cell line and Jurkat cell line. In summary, these evidences demonstrate that TANCR is a noncoding RNA which positively regulates TRAIL expression in γδ T cells in responding to IPP treatment.

## Discussion

Noncoding RNAs, especially lncRNA, have recently regarded as critical regulators in the immune system [26]. Developments in NGS have revealed that most DNA in the genome encodes lncRNAs [27, 28], however, the functions of lncRNAs remain to be determined. Hence, exploring the roles of lncRNA in the immune system may help us clearly understand the role that lncRNAs have. γδ T cells are well known because of their key roles in defense against various viral and bacterial infections [29–31]. IPP is an intermediate produced from mevalonate that can selectively activate and expand human γδ T cells [23, 24, 30]. NcRNA studies in the immune system are currently focused on immune cells such as natural killer (NK) cells, T helper cells, and B cells [32–35], while less is known about lncRNA regulation in γδ T cells. We therefore sought to investigate the function of lncRNAs in γδ T cell biology. In this study, γδ T cells were activated and expanded by IPP (Fig. 2). MACS was performed to purify γδ T cells. Flow cytometry was then conducted to detect the percentage of purified γδ T cells (Fig. 2). NGS and bioinformatic analysis were performed to figure out the differentially expressed mRNA and lncRNA. Go and KEGG analyses were conducted to predict the biological function and pathways of differentially expressed genes (Fig. 3). Since cis-regulation is one typical regulation mechanism in lncRNA regulation network, [13, 15] so we then predicted the potential lncRNA–mRNA pairs that are regulated in cis based on the differentially expressed genes and we summarized the top 15 upregulated and downregulated lncRNA–mRNA pairs in Table 1. We then selected several genes that were differentially expressed in the sequencing results and performed qRT-PCR to verify the accuracy of sequencing in activated γδ T cells. qRT-PCR results showed that the RNA expression of selected genes was consistent with the sequencing results (Fig. 5a, b), indicating that the sequencing data were valid. We focused on the regulation of lncRNA on the cytokines that are secreted by activated γδ T cells due to their important roles in γδ T cells cytotoxicity in this study. Interestingly, we found one lncRNA named TANCR (we defined the name according to a previously published paper [25]) that regulates TRAIL expression during γδ T cells activation. TANCR is a lncRNA that located downstream of the TRAIL gene (Fig. 6a). TRAIL is a cytokine that is expressed on the surface of many immune cells, including NK cells and T lymphocytes [36]. TRAIL is regarded as important antitumor cytokine because it can induce target cell apoptosis by binding to the receptors tumor necrosis factor related apoptosis-inducing ligand 1 (TRAILR1, also known as DR4) and tumor necrosis factor related apoptosis-inducing ligand 2 (TRAILR2, also known as DR5) that are expressed on the surface of target cell [37–39]. To further confirm TANCR and TRAIL expression in fresh and activated γδ T cells, we collected seven blood samples and isolated PBMCs samples respectively. After IPP treatment for two weeks, RT-PCR was performed and the results revealed that the expression of TANCR and TRAIL were both upregulated in IPP-expanded γδ T cells from seven samples (Fig. 5c, d). In addition, Spearman analysis showed a correlation between TANCR and TRAIL (Fig. 5e). These results indicate that TANCR might regulate TRAIL expression during the activation of γδ T cells. We also found that TANCR expression was much higher in γδ T cells than in L-02 cell line, Jurkat cell line, K562 cell line, A549 cell line, and HEK293T cell line (Fig. 5f), which emphasizes the potential function of TANCR in γδ T cells. We then isolated nuclear and cytoplasmic RNA from HEK293T cells and Jurkat cells and found that TANCR localized both in nucleus and cytoplasm (Fig. 6b, c).

An TANCR overexpression vector was constructed and transfected into HEK293T cells, we found both of the TRAIL mRNA and protein were increased (Fig. 7). TANCR was located both in the nucleus and cytoplasm, while it was much more highly expressed in the cytoplasm than in the nucleus (Fig. 6b, c). Therefore, A mixture of siRNA and ASO were then used to knockdown TANCR expression in the cytoplasm and nucleus in HEK293T cell line. Downregulation of TANCR with siRNA and ASO reduced the expression of TRAIL both at the mRNA and protein level (Fig. 8b, c). To further confirm the regulation between TANCR and TRAIL, we used CRISPR/Cas9 system to knock out TANCR in Jurkat cells and primary γδ T cells. A plasmid containing the cas9 protein was first transduced into cells using a lentivirus followed by guide RNAs. With the silencing of TANCR, TRAIL mRNA and protein were both decreased in Jurkat cells and primary γδ T cells (Figs. 8f, h, 9c, e), consistent with the results from HEK293T cell line. Hence, these evidences demonstrate that we identified a lncRNA named TANCR that is predominantly expressed in the cytoplasm and positively regulates TRAIL expression in cis in activated γδ T cells. In addition, our data revealed that the lncRNA and mRNA expression in IPP-expanded γδ T cells is significantly different from fresh γδ T cells controls. We also predicted the potential effect of these differentially expressed genes and the interaction between lncRNA and mRNA that can be used for further studies.

In this study, we found TANCR is highly expressed in activated γδ T cells, which means that TANCR is important in regulating γδ T cells activation by promoting TRAIL expression. The upregulation of TANCR and TRAIL in IPP-activated γδ T cells from seven samples indicates that TANCR might also be a potential activation marker for γδ T cells. However, this hypothesis should be confirmed by testing a larger number of samples. The regulation between TRAIL and TANCR in NK cells should be validated due to TRAIL is also considered as a NK cell cytokine [40, 41]. The current study focused on regulation occurring in cis, while lncRNAs can also regulate gene expression in trans or directly interact with proteins [13]. These interactions should be considered as important mechanisms during lncRNA regulation that can be studied in the immune response. γδ T cells are distributed in many lymphoid tissues [42], and the discovery of tissue-specific lncRNAs may be important to better understanding γδ T cells biology. Meanwhile, there are various cytokines that are expressed by γδ T cells, and the functions of lncRNAs in regulating other cytokines should also be identified. However, γδ T cells make up a small part of the immune system, and there are numerous immune cells during the immune response. It is therefore necessary to identify the function of lncRNAs in other immune cells, which will help us construct a precise lncRNA regulation network in the immune system.

## Conclusion

In summary, we have provided evidence that the lncRNA TANCR, expressed predominantly in the cytoplasm, positively regulates TRAIL expression in γδ T cell, which demonstrate an enhanced function of lncRNAs in γδ T cell activation.

## Material and methods

### Study subject

All the samples used in this study were obtained from West China Second University Hospital with informed consent from all participants, following the Ethics Committee of Sichuan University.

### Cell isolation and γδ T cells preparation

Ficoll (TBD, Tianjin, China) was used to isolate PBMCs. Ficoll was pipetted into a 50 ml tube, and the blood was added to the tube gently. Centrifuge the 50 ml tube for 20 min at 800*g*. Pipette the cell layer (PBMCs) between blood and Ficoll into a clean 50 ml tube. Cold phosphate buffer saline (PBS) was used to wash the cells twice and the cells were centrifuged at 600*g* for 5 min. The isolated PBMCs were exposed to IPP (6 μg/ml) added medium for 3 days and then cultured in medium containing IL-2 (Invitrogen, Carlsbad, CA, USA) up to two weeks. Fresh medium was added every 3 days [43]. γδ T cells were finally purified with an Anti-TCR gamma delta Micro-Bead Kit (Miltenyi Biotec, Germany) from IPP treated PBMCs according to the manufacturer’s instructions.

### Cell culture and viral infection

DMEM medium (Invitrogen, Carlsbad, CA, USA) supplemented with 10% fetal bovine serum (FBS, Gibco, Carlsbad, CA, USA) was used to culture HEK293T cells. Jurkat cells and primary γδ T cells were cultured in RPMI medium (Invitrogen, Carlsbad, CA, USA) with 10% FBS. All the medium was supplemented with 100 μg/ml streptomycin and 100 U/ml penicillin (Gibco). Cells were cultured at 37 °C in a 5% CO_2_ incubator (Sanyo, Osaka, Japan). siRNAs were used to silencing TANCR expression in HEK293T cells. A negative control siRNA (NC siRNA) was used. siRNA/ASO was transfected using Lipofectamine™ RNAi MAX Transfection Reagent (Invitrogen, Cartsbad, USA). To knock out TANCR in Jurkat cells and γδ T cells, a vector containing TANCR guide RNAs and plasmid containing cas9 protein were packaged in HEK293T cells respectively. Jurkat cells and γδ T cells were firstly infected with cas9 lentivirus and selected by G418. TANCR guide RNA lentivirus was then transduced in these cells [44].

### RNA-Seq

RNA was extracted from IPP-expanded and fresh γδ T cells using Trizol (Invitrogen, Cartsbad, USA), followed by ribosomal RNA removal using Ribo-Zero™ rRNA Removal Kit (Epicentre, Madison, WI, USA). A strand specific cDNA library was constructed using TruSeq® Stranded kit (Illumina, Madison, WI, USA). RNA sequencing was conducted by an Illumina Hi Seq 4000 platform (Illumina, San Diego, CA, USA) by Novogene. The sequenced reads were aligned to the human reference genome with HISAT [45] and PossionDis [46] was used to select differential expressed lncRNA/mRNA (fold change < ‒ 2 or > 2 and FDR p value < 0.05).

### Flow cytometry

Cells were blocked with 5% BSA diluted in PBS for 20 min and then stained with the following surface antibodies: anti-CD3, anti-TCR γδ, and anti-TRAIL for 30 min. Cold PBS was then used to wash the cells three times. Flow cytometry (BD FACSCelesta) was used to detect the cells, and FlowJo software was used to analyze the data. Antibodies used were obtained from Biolegend (San Diego, USA). Antibodies were obtained from BD.

### Nuclear and cytoplasmic RNA isolation

The nuclear and cytoplasmic RNA was isolated using protocol from Cold Harbor Laboratory [47]. Briefly, HEK293T cells and Jurkat cells were collected from tissue culture dishes and washed by cold phosphate-buffered saline (PBS) for three times. Then the cells were resuspended in cold disruption buffer (1.5 mM MgCl_2_, 10 mM KCl, 20 mM Tris–HCl, pH = 7.5, 1 mM DTT). Cells were then incubated on ice for 10 min. Dounce homogenizer was used to disrupt the cell membrane. The microscope was used to ensure that 90% of the cell membrane was broken during homogenate. The nuclei should not be broken. The homogenate was then transferred to a fresh tube and Triton X-100 was added to make a final concentration of 0.1%. The tubes were inverted four to five times. The nuclear and cytoplasmic fractions were separated by centrifuging the homogenate at 1500*g* for 5 min. The supernatant was transferred to a fresh tube without disturbing the nuclear pellet. RNA was extracted using Trizol according to the manufacturer’s instruction (Invitrogen, Cartsbad, USA).

### RNA extraction and qRT-PCR

Trizol was used to extract RNA. Reverse transcription was conducted with SuperScript™ III First-Strand Synthesis System (Invitrogen, Cartsbad, USA) according to the manufacturer’s instructions. PowerUp™ SYBR™ Green Master Mix was used to perform qRT-PCR on an Applied Biosystems 7500 (Life technologies, Cartsbad, USA). The qRT-PCR results were normalized by internal control GAPDH. Sequence of primers is shown in Table 2. Primers were synthesized by GENEWIZ (Suzhou, China).

**Table 2:** Real time-PCR primers

Name	Sequence
GAPDH-forward	GAGTCAACGGATTTGGTCGT
GAPDH-reverse	TTGATTTTGGAGGGATCTCG
TRAIL-forward	CAAGCCCCATCAAGGACTGG
TRAIL-reverse	GAAGGTAGCGTGTGGGGATT
TANCR-forward	TCTTGGCCTCCAAATTGTCACT
TANCR-reverse	TCAAACTTCCCAAGTGTGCTT
U6-forward	CTCGCTTCGGCAGCACATATACT
U6-reverse	ACGCTTCACGAATTTGCGTGTC
Cas9-forward	CATCGAGCAGATCAGCGAGT
Cas9-reverse	CGATCCGTGTCTCGTACAGG

### Western blot

Cells were harvested using lysis buffer (Beyotime). Proteins were separated by a 12% SDS-PAGE gel and then transferred to a nylon membrane. 5% skim milk diluted in PBS-T (1% of Tween-20 in PBS) was used to block the membrane at room temperature for 1 h. Primary antibody diluted with PBS-T was incubated with the membrane at 4 °C overnight (TRAIL, ProteinTech) and glyceraldehyde-3-phosphate dehydrogenase (GAPDH, Abcam) antibody with a dilution of 1:1000). After the membrane was washed with PBS-T three times for 5 min, the membrane was then incubated with horseradish peroxidase-conjugated secondary antibody (CST) diluted with PBS-T for 1 h at room temperature. Pierce™ ECL Western Blotting Substrate (Thermo, Cartsbad, USA) was used to detect the signal by using a Chemidoc imaging system (Bio-Rad Laboratories) [48].

## Data Availability

The data that support the findings of this study are available from the corresponding author upon request.

## Abbreviations

lncRNA: Long noncoding RNA
PBMCs: Peripheral blood mononuclear cells
TCR: T cell receptor
TRAIL: Tumor necrosis factor related apoptosis-inducing ligand
TANCR: Tumor necrosis factor related apoptosis-inducing ligand activated noncoding RNA.
IPP: Isopentenyl pyrophosphate.
Fas: Factor associated suicide
FasL: Factor associated suicide ligand
IFN-γ: Interferon-γ
APCs: Antigen presenting cells
IL-10: Interleukin 10
TGF-β: Transforming growth factor β
